# Analysis of AT7519 as a pro-resolution compound in an acetaminophen-induced mouse model of acute inflammation by UPLC-MS/MS

**DOI:** 10.1186/s12950-023-00345-y

**Published:** 2023-06-08

**Authors:** Jennifer A. Cartwright, Joanna P. Simpson, Natalie Z. M. Homer, Adriano G. Rossi

**Affiliations:** 1 University of Edinburgh Centre for Inflammation Research, Institute for Regeneration and Repair, 4-5 Little France Drive, Edinburgh BioQuarter, Edinburgh, Midlothian EH16 4UU United Kingdom; 2 Centre for Regenerative Medicine, Institute for Regeneration and Repair, University of Edinburgh, 4-5 Little France Drive, Edinburgh BioQuarter, Edinburgh, Scotland EH16 4UU UK; 3 Mass Spectrometry Core, Edinburgh Clinical Research Facility, Centre for Cardiovascular Sciences, Queen’s Medical Research Institute, University of Edinburgh, 47 Little France Crescent, Edinburgh, EH16 4TJ UK

**Keywords:** Mass spectrometry, Neutrophil, Hepatic, Paracetamol

## Abstract

**Background:**

Uncontrolled inflammation contributes to the progression of organ damage in acute conditions, such as acetaminophen-induced acute liver injury (APAP-ALI) and there are limited treatments for this condition. AT7519, a cyclic-dependent kinase inhibitor (CDKI), has been used successfully in several conditions, to resolve inflammation and return tissue homeostatic functions. AT7519 has not been assessed in APAP-ALI and its effect on APAP metabolism is unknown. Targeted chromatography and mass spectrometry can be used to assess multiple compounds simultaneously and this approach has not been applied yet to measure APAP and AT7519 in a mouse model.

**Results:**

We show an optimised simple and sensitive LC–MS/MS method for determining concentrations of AT7519 and APAP in low volumes of mouse serum. Using positive ion mode electrospray ionisation, separation of AT7519 and APAP and their corresponding isotopically labelled internal standards [^2^H]_8_-AT16043M (d8-AT7519) and [^2^H]_8_-APAP (d4-APAP), was achieved on an Acquity UPLC BEH C18 column (100 × 2.1 mm; 1.7μm). A gradient mobile phase system of water and methanol was delivered at a flow rate of 0.5 mL/min with a run time of 9 min. Calibration curves were linear, intra-day and inter-day precision and accuracy were acceptable and the covariates of all standards and quality control replicates were less than 15%. The method was successfully applied to evaluate AT7519 and APAP levels 20 h post AT7519 (10 mg/mg) in C57Bl6J wild type mouse serum treated with either vehicle or APAP. Serum AT7519 was significantly higher in mice that had received APAP compared to control, but there was no correlation between APAP and AT7519 quantification. There was also no correlation of AT7519 and hepatic damage or proliferation markers.

**Conclusion:**

We optimised an LC–MS/MS method to quantify both AT7519 and APAP in mouse serum (50 µL), using labelled internal standards. Application of this method to a mouse model of APAP toxicity proved effective in accurately measuring APAP and AT7519 concentrations after i.p. dosing. AT7519 was significantly higher in mice with APAP toxicity, indicating hepatic metabolism of this CDKI, but there was no correlation with markers of hepatic damage or proliferation, demonstrating that this dose of AT7519 (10 mg/kg) does not contribute to hepatic damage or repair. This optimised method can be used for future investigations of AT7519 in APAP in mice.

## Background

Improving inflammation resolution is a key area of ongoing research to allow for damaged tissues to repair and recover towards pre-injury or adaptive homeostasis [1]. Inflammation is a controlled and appropriate response to tissue injury and in the majority of cases is followed by a tightly orchestrated and complex resolution process [2–4]. There are, however, a myriad of conditions where inflammation is overwhelming, leading to acute tissue damage, irreversible organ changes and failure, or to long term chronic inflammation, resulting in sever patient morbidity and high health care costs [5–7].

Several mechanisms have been exploited in an attempt to change the course of this uncontrolled inflammation, some more successful than others. Our laboratory has focussed on pharmacological mechanisms that target the innate immune system component of the inflammatory response with drugs that can promote granulocyte clearance [8]. One class of drugs that has been shown to do this is cyclic-dependent kinase inhibitors (CDKIs), and since this application was first identified in 2006, drugs of this class have been applied successfully to resolve inflammation in several models [9, 10]. As well as being used in many models, this drug class is being trialled and considered in several human conditions [11–14].

There are many conditions of both chronic and acute inflammation in which CDKIs have not been assessed. One condition, for which therapeutic advances have not occurred since the addition of N-acetylcysteine in the 1970s, is acetaminophen (APAP)-induced acute liver injury (APAP-ALI) [15, 16]. This toxicity leads to acute tissue necrosis and a profound secondary innate immune response of infiltrating neutrophils and monocytes [17, 18]. Modulating innate immune responses with monocyte cell therapy is ongoing [19], but targeting the inflammatory neutrophil component requires further investigation. Neutrophils infiltrate the liver rapidly after APAP-ALI, increasing to high levels at 12 and 24 h and have been shown to have a detrimental role [20–22], as is seen in other conditions [23]. We therefore aimed to assess the use of a CDKI, AT7519 in this condition.

AT7519 is beneficial in several animal models of inflammation and promotes human airway granulocyte depletion [8, 11], but has not yet been evaluated with diseases of the liver, a highly regenerative organ, nor in conjunction with APAP. The available literature of analytical methods for the quantitation of AT7519 is limited [24–26], and currently no studies have quantified both APAP and AT7519 simultaneously, particularly in small volumes of serum typically obtained from systemically unwell mice. We therefore report the development and assessment of an LC–MS/MS method for the simultaneous quantitation of APAP and AT7519 in small volumes (~ 50 µL) of mouse serum.

## Materials and Methods

### Chemicals and reagents

**Fig. 1:**
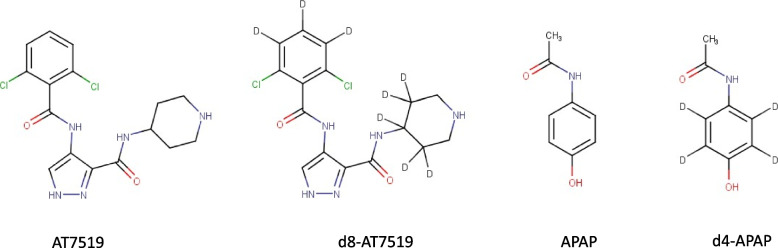
Structures of AT7519, acetaminophen (APAP) and their corresponding isotopically labelled standards d8-AT7519 and d4-APAP

AT7519 and a corresponding deuterated standard [^2^H]_8_-AT16043M (d8-AT7519) were supplied as a gift by Astex Pharmaceuticals (Cambridge, UK), as methane sulfonic acid salts. APAP was purchased from Apollo (Denton, Manchester, UK) and a [^2^H]_8_-APAP (d4-APAP) stock solution was purchased as a certified reference material at 100 µg/mL in methanol from Cerilliant® (Merck, Watford, UK). The structure of APAP, AT7519 and the corresponding isotopically labelled internal standards are shown in Fig. 1.

Water (LC–MS grade), methanol (LC–MS grade), 2-propanol (LC–MS grade) and acetonitrile (HPLC grade) were purchased from VWR. Formic acid (LC–MS grade) was purchased from Fisher Scientific (Loughborough, UK) and bovine serum albumin 5% in 0.85% sodium chloride from Sigma Aldrich (Dorset, UK).

### Instrument and chromatographic conditions

Samples, held at 10 °C in the autosampler, were injected (20 µL) into the chromatographic system of a Waters Acquity Classic UPLC unit with an Acquity UPLC BEH C18 (2.1 × 100 mm 1.7 µm; Waters, Wilmslow, UK) column maintained at 45 °C. The mobile phase consisted of 0.1% formic acid in water (A) and 0.1% formic acid in acetonitrile (B) at a flow rate of 0.5 mL/min. Gradient elution was achieved with a total run time of 9.0 min. The gradient conditions were as follows: An initial hold of 5% B for 1.5 min, with an increase up to 95% B over 5 min, held at 95% B for 2 min, followed by a 2 min equilibration at 5% B. Optimised mass spectrometry parameters of de-clustering potential, entrance potential, collision energy and collision cell exit potential for all analytes (APAP and AT7519 and internal standards d8-AT7519 and d4-APAP) are shown in Table 1.

**Table 1:** Optimized mass spectrometry settings on a QTrap 5500 mass spectrometer for APAP, AT7519 and isotopically labelled internal standards, operated in positive mode. Voltage (V). All analytes and standards were infused and analysed in positive ion mode

Analyte		Precursor ion* (m/z)*	Product ion* (m/z)*	De-clustering potential (V)	Entrance potential (V)	Collision Energy (V)	Collision exit potential (V)
AT7519	[M + H]^+^	382.1	84.1	66	9	29	10
d8-AT7519	[M + H]^+^	390.1	89.1	66	9	29	10
APAP	[M + H]^+^	152.0	110.1	81	9	23	12
d4-APAP	[M + H]^+^	156.0	114.0	81	9	23	12

Mass analysis was performed on a QTrap 5500 triple quadrupole mass spectrometer (AB Sciex, Warrington, UK) operated in positive ion electrospray mode (5.5 kV, 500 °C, ion source gas 1 at 60 psi and gas 2 at 40 psi). In multiple reaction mode, transitions were identified (see results), and the peak area ratio of APAP/d4-APAP and AT7519/d8-AT7519 were used to calculate the amount of APAP and AT7519 by linear regression analysis of a calibration curve (0.5–50 ng/mL). LC–MS/MS data were evaluated by investigators blinded to the sample treatment.

### Preparation of drug solutions, calibrations standards and quality control samples

Stock solutions of APAP, AT7519 and d8-AT7519 were prepared in methanol at a concentration of 1 mg/mL. d4-APAP stock solution was purchased as a certified material at 100 µg/mL in methanol from Cerilliant, UK. A working stock solution containing both APAP and AT7519 was prepared at 50 µg/mL, and further diluted to 0.5, 1, 2.5, 5, 10, 25, 45, and 50 ng/mL. A working internal standard solution containing d4-APAP and d8-AT7519 (50 ng/mL) was prepared in methanol. All standards were prepared in 4% BSA. Each sample was enriched with 1 ng working internal standard. Quality control samples were prepared in 4% BSA solution at four concentrations: LLOQ-QC (0.5 ng/mL), Low-QC (0.75 ng/mL), Mid-QC (5 ng/mL) and the High-QC (40 ng/mL). To each QC sample, 1 ng working internal standard was added. All standards, sample and QC-samples were defrosted and mixed for 10 min at 400 rpm before extraction. All standard solutions were stored at –20◦C and brought to room temperature before use.

### Sample preparation

Sample and calibration standard extractions were automated on a 96-well protein precipitation (PPT +) plate (Biotage, Sweden). Frozen samples were thawed at room temperature and 50 µL of serum was transferred to a 2 mL 96-well plate. Standards were prepared in 50 µL of 4% BSA. Samples and standards were enriched with the working internal standard (1 ng d4-APAP and d8-AT7519) and diluted in water (1:1). Samples and calibration standards were transferred to an Extrahera liquid handling robot (Biotage, Uppsala, Sweden). The liquid handling robot added acetonitrile (400 μL) to each well then transferred the samples to a PPT + plate (2 mL, Biotage) for extraction. The eluate was pulled through under positive pressure into a 2 mL deep well 96 well collection plate (2 mL, Waters, UK).

The eluate was reduced to dryness under nitrogen stream at 40 °C on an SPE Dry 96 Dual Sample Concentrator (IST, UK). Once dry, samples were resuspended in water:methanol (90:10 v/v; 100 µL) and sealed with a Zone-free 96 well plate sealing film (VWR). The samples were aggregated in a plate shaker (Thermoshaker 3005 GFL, ThermoScientific, UK) for 10 min at 600 rpm prior to analysis by LC–MS/MS.

### Assessment of accuracy and precision

#### Accuracy

To assess the accuracy of the method, QC samples were prepared using 4% BSA solution and spiked with known amounts of AT7519 and APAP. The QC samples were analysed against the calibration curve. Here, the accuracy of both the standards and QC samples are reported as a percentage of the nominal value (%NOM). The %NOM is the calculated concentration expressed as a percentage of the nominal concentration using the following equation.

$$accuracy\left(\%NOM\right)=\frac{calculated\,concentration}{nominal\,concentration}\;\times\;100$$

To assess the accuracy of the standard curve, the concentrations of each standard were back calculated by plotting the peak area ratios of AT7519 and APAP to the internal standards d8-AT7519 and d4-APAP respectively. A weighted (1/x^2^) least-squares linear regression analysis was performed using Analyst® 1.7.1 software. Acceptance criteria for accuracy of both standards and QC samples was set at ± 15%, except the LLOQ standard and LLOQ QC samples, which had an acceptance of ± 20%.

#### Intra batch (within-run) accuracy

To assess within run (intra-batch) accuracy, alongside the standard curve; four QC sample replicates per level (LLOQ, Low, Medium and High); 0.5, 0.75, 5.0 and 40.0 ng/mL) were included. The following equation was used to calculate the within-run (intra-batch) accuracy of each QC level.

$$\mathrm{intra\,batch\,accuracy\,of\,QC\,sample}=\frac{Mean\,calculated\,concentration\,of\,QC\,sample\,in\,1\,batch}{nominal\,concentration\,of\,QC\,sample} \times 100$$

#### Inter batch (between run) accuracy

To assess the between run accuracy (inter-batch), three batches were analysed over two days. Each batch included a standard curve, and four QC sample replicates per level. The inter-batch accuracy of each QC level was calculated using the following equation.

$$\mathrm{inter\,batch\,accuracy\,of\,QC\,sample}=\frac{Mean\,calculated\,concentration\,of\,QC\,sample\,in\,3\,batches}{nominal\,concentration\,of\,QC\,sample} \times100$$

### Precision

#### Intra-batch precision of QC samples

To assess the within run (intra-batch) precision, the coefficient of variation (%CV) of 6 QC levels was calculated in a single batch, using the standard deviation (SD) and arithmetic mean in the following equation, with an acceptance criterion of ± 15%.

$$\mathrm{Intra\,batch\,precision }(\mathrm{\%CV})=\frac{SD\,of\,calculated\,concentration\,of\,QC\,samples\,in\,1\,batch}{mean\,concentration\,of\,QC\,samples\,in\,1\,batch} \times100$$

#### Inter-batch precision of QC samples and standards

The between run (inter-batch) precision, was calculated for both the standards (*n* = 3) and the QC samples (*n* = 12). The %CV of each standard and QC level was calculated across three batches, using the following equation.

$$\mathrm{Inter\,batch\,precision }(\mathrm{\%CV})=\frac{SD\,of\,calculated\,concentration\,of\,QC\,samples\,in\,3\,batches}{mean\,concentration\,of\,QC\,samples\,in\,3\,batches} \times 100$$

### Specificity and selectivity

Selectivity was assessed by analysing serum samples from mice treated with APAP or AT7519 and vehicle treated mouse samples spiked with either compound separately. Specificity was tested by analysing five serum samples from mice not treated with APAP or AT7519, and five samples from mice that received either AT7519 or APAP.

### Recovery and Matrix effects

The recovery of both analytes extracted from BSA using PPT + was assessed. Standards were prepared at 50 ng/mL in 4% BSA (100 μL). Peak areas of standards extracted from BSA (pre-spike, *n* = 3) were compared to standards spiked into BSA extracts post extraction (post-spike, *n* = 3). The % recovery was calculated using the following equation.

$$\mathrm{\%\,recovery}=\frac{mean\,peak\,area\,of\,analyte\,in\,pre\,spike}{mean\,peak\,area\,of\,analyte\,in\,post\,spike} \times100$$

The matrix effect of BSA for both analytes was calculated using analyte peak area in post spike standards (50 ng/mL) (*n* = 30) in BSA extracts, compared to peak area in a pure solution (*n* = 3) of the same concentration. The equation to calculate matrix effect is shown below.

$$\mathrm{matrix\,effect}=\frac{mean\,peak\,area\,of\,analyte\,in\,post\,spike}{mean\,peak\,area\,of\,analyte\,pure\,solution} \times 100$$

### Stability

Stability of extracted samples stored at 10 °C in the autosampler was assessed by immediate injections, followed by a second injection of standards and QCs (LLOQ, Low, Mid and High) after 48 h in the autosampler and the peak areas and concentrations compared with analysis at 0 h.

### In vivo* studies*

#### Animal studies

Ten week old C57Bl6J male mice (purchased from Charles River, UK) were acclimatised to unit conditions for 1 week prior to experiments. Mice were housed in groups of five in an individual ventilated cage system, and synchronized to a 10–14 h dark/light cycle with access to food and water ad libitum. All animal experiments were undertaken in accordance with criteria outlined in a license granted under the Animals (Scientific Procedures) Act 1986, and approved by the University of Edinburgh Animal Ethics Committee.

Mice were fasted 12 h prior to a 350 mg/kg injection of APAP in sterile saline, (PanReac Applichem) or vehicle (sterile saline). Standard chow and mash were returned to mice 20 min after injection. To all mice, AT7519 dissolved in sterile saline at 2.5 mg/ml, dosed at 10 mg/kg or vehicle was injected at 16 h post APAP injection and whole blood was collected at 36 h post APAP injection from the caudal vena cava. Whole clotted blood samples were centrifuged at 5,000 g for 5 min, from which the serum supernatant was collected. This process of centrifugation of the collected serum was repeated to ensure no red blood cell contamination. Serum was stored at -80 °C until analysis.

### Serum chemistry evaluation

Serum chemistry was performed utilizing a commercial kit (Alpha Laboratories) for alanine aminotransferase (ALT) [27], adapted for use on a Cobas Fara centrifugal analyser (Roche Diagnostics).

### Immunohistochemistry

FFPE 4 µm thick sections were dewaxed and rehydrated before heat mediated antigen retrieval for 15 min in either TrisEDTA (Ph9), then permeabilised in PBS 0.1% Tween 20 (PBST). Sections were blocked for 30 min with Protein Block (Spring Bio), then incubated with the primary antibodies HNF4α (1:200, Perseus Proteomics, PP-H1415-00), Ly6G (1:500, Biolegend, 127,602) and minichromosome maintenance complex component 2 (MCM2) (1:200, Cell Signalling, 4007S) overnight at 4 °C. Following washing secondary antibodies; Donkey, anti-goat 555, (Invitrogen a32816), donkey anti-rabbit 647 (Invitrogen A32795) and donkey anti-rat 488 (Invitrogen A21208) were applied for 1 h, with DAPI (1:1000) and mounted with flouromount (Southern Biotech).

### Microscopy and Image analysis

Bright field images were also acquired on a Vectra® Polaris™ multi spectral slide scanner (PerkinElmer) and fluorescent images collected on an Operetta CLS High Content Analysis System (PerkinElmer). Numbers and percentage of MCM2 positive hepatocytes (HNF4 α + , DAPI +) cells were analysed using Columbus™ software (Perkin Elmer). On H&E stains, necrotic areas were identified through a lack of intact hepatocyte nuclei, disordered tissue structure, and hepatocyte ballooning. These parameters were used with spectral unmixing to train tissue analysis software, inForm 2.4 (Perkin Elmer) for quantification.

### Data analysis

LC–MS/MS data was collected using Analyst® 1.7.1 software and the targeted data and linear regression were evaluated using MultiQuant 3.1.3 (AB Sciex, UK), while assay validation precision and accuracy was calculated in Microsoft Excel ® 2016. Graph Pad Prism 8 was used for statistical analysis of mouse serum APAP and AT7519 concentrations and correlations with tissue damage and repair markers. Testing for normality was completed with a Shapiro–Wilk test. Data without a gaussian distribution and difference in variation were assessed with a Kolmogorov–Smirnov test.

## Results

### Chromatographic and mass spectrometric conditions

**Fig. 2:**
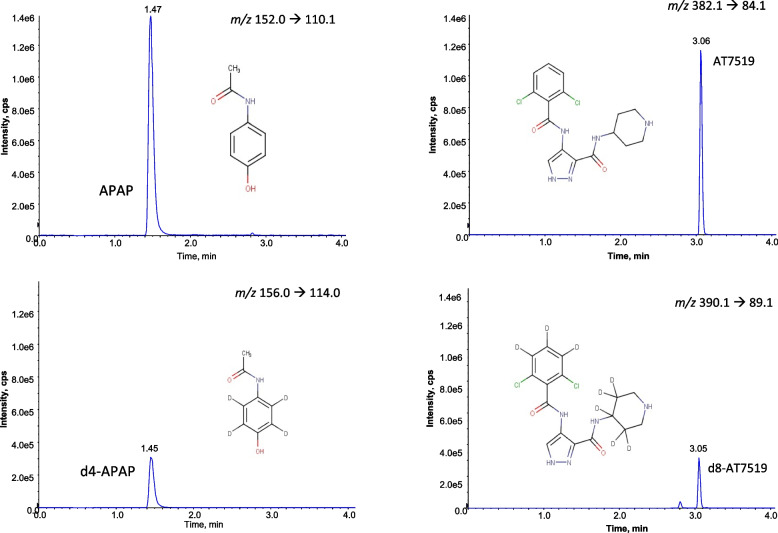
Extracted ion chromatograms of an analytical standard at 5 ng, showing retention time of AT7519 and APAP and the isotopically labelled internal standards, d8-AT7519 and d4-APAP following separation on a Waters Acquity Class UPLC system on a BEH C18 column (100 × 2.1 mm; 1.7 µm) and a mobile phase system of water and methanol (0.5 mL/min; 50 °C) followed by positive ionisation mode electrospray ionisation and multiple reaction mode analysis on a QTrap 5500 mass spectrometer

In positive ionisation multiple reaction mode (MRM), the major transitions were identified as *m/z* 382.1→89.1 for AT7519, 390.1→136.0 for AT7519-d8, 152.0→110.1 for APAP and 156.0→114.0 for APAP-d4 (Table 1). Ions were monitored at retention times of 1.70, 1.46, 3.06 and 3.05 min for APAP, d4-APAP, AT7519 and d8-AT7519 respectively (Fig. 2).

### Linearity, precision and accuracy

#### Assessment of linearity and inter-batch precision and accuracy of calibration curves

Calibration curves were assessed for both AT7519 and APAP and found to be linear from 0.5 ng/mL to 50 ng/mL for both compounds in all 3 batches. All standards met the acceptance criteria for inter-batch precision and accuracy, with a % NOM value within ± 15% of the nominal concentration, and ± 20% for the LLOQ standard. When assessing precision, the %CV of standards across three batches was found to be less than 15%. Covariance for each standard ranged from 1.84- 13.51% (APAP) and 3.00–5.86% (AT7519) (Table 2).

**Table 2:** Inter-batch precision (%CV) and accuracy (%NOM) assessment for APAP and AT7519 standards analysed in 3 batches, over two days

nominal concentration (ng/mL)	N	APAP			AT7519		
		**mean calculated concentration (ng/mL)**	**%CV**	**%NOM**	**mean calculated concentration (ng/mL)**	**%CV**	**%NOM**
**0.5**	**3**	0.53	3.1	105.60	0.54	5.86	108.53
**1**	**3**	0.86	13.51	85.70	0.97	3.32	96.60
**2.5**	**3**	2.68	12.11	107.39	2.36	4.84	94.21
**5**	**3**	5.05	2.29	101.02	4.84	3.70	96.83
**10**	**3**	9.93	3.16	99.33	9.50	4.86	94.96
**25**	**3**	24.33	3.03	97.30	25.33	4.16	101.30
**45**	**3**	45.11	1.84	100.25	46.14	3.00	102.54
**50**	**3**	51.70	2.50	103.40	52.48	3.82	104.96

### Assessment of intra and inter-batch precision and accuracy of QC samples

QC samples were assessed at four concentrations (0.5, 0.75, 5.0 and 40 ng/mL, *n* = 4 at each concentration) over one batch (intra-batch assessment) and three batches analysed over two days (inter-batch assessment). Intra and inter batch assessment of accuracy and precision for both analytes (APAP, Table 3 and AT7519, Table 4) were found to meet the acceptance criteria with an average % NOM value within ± 15% of the nominal concentration, and a %CV of less than 15.

**Table 3:** Intra and inter-batch precision and accuracy assessment of APAP measurements using QC samples prepared in Bovine Serum Albumin, (LLOQ – lower limit of quantitation, QC – quality control)

**QC Level**	**N**	**Nominal concentration (ng/mL)**	**Intra CV (%)**	**% Norm Intra**		**Inter CV (%)**	**% Norm Inter**
LLOQ QC	12	0.50	8.90	102.60		12.64	93.97
Low QC	12	0.75	2.70	108.29		12.63	95.44
Mid QC	12	5.00	1.98	93.91		3.85	96.27
High QC	12	40.00	2.07	97.43		2.22	96.93

**Table 4:** Intra and inter-batch precision and accuracy assessment of AT7519 measurements using QC samples prepared in Bovine Serum Albumin (LLOQ – lower limit of quantitation, QC – quality control)

QC Level	N	Nominal concentration (ng/mL)	Intra CV (%)	% Norm Intra	Inter CV (%)	% Norm Inter
LLOQ QC	12	0.50	2.86	97.50	7.94	107.58
Low QC	12	0.75	2.09	101.04	5.64	107.54
Mid QC	12	5.00	1.80	93.11	3.31	96.04
High QC	12	40.00	1.40	99.28	3.42	102.56

### Specificity, selectivity, recovery and matrix effects.

#### Specificity and selectivity

**Fig. 3:**
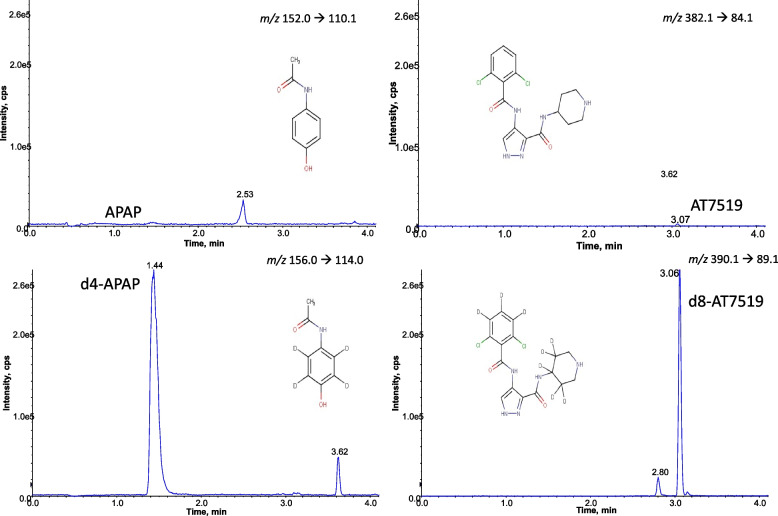
Extracted ion chromatogram of a plasma sample (50μL) from a mouse not treated with AT7519 or APAP, separated on a BEH C18 (100 × 2.1 mm; 1.7 μm) column followed by MRM analysis on a QTrap 5500. APAP – acetaminophen, AT7519 – a cyclic kinase inhibitor, d4-APAP – deuterated acetaminophen, d8-AT7519 – deuterated AT7519

**Fig. 4:**
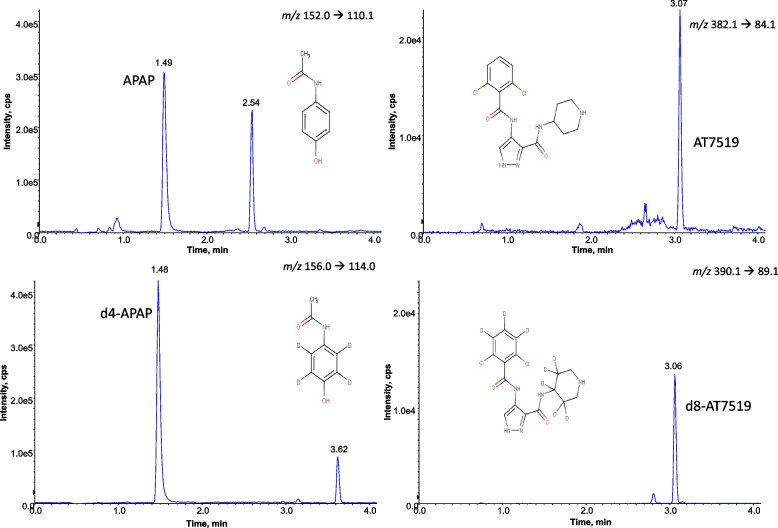
Extracted ion chromatogram of a plasma sample (50 μL) from a mouse treated with AT7519 and APAP, separated on a BEH C18 (100 × 2.1 mm; 1.7 μm) column followed by MRM analysis on a QTrap 5500. APAP – acetaminophen, AT7519 – a cyclin-dependent kinase inhibitor, d4-APAP – deuterated acetaminophen, d8-AT7519 – deuterated AT7519

No chromatographic peaks were detected at the relevant mass transitions at 1.70, 1.46, 3.06 and 3.05 min for APAP, d4-APAP, AT7519 or d8-AT7519 in control mouse serum extracted from 5 individuals, or in BSA blanks (*n* = 5). The method was able to identify both compounds in the same sample and quantify the compounds in the presence of matrices. An example extracted ion chromatogram for APAP and AT7519 from control (untreated) and poistive (treated) mouse serum, and BSA blank are shown in Fig. 3 and Fig. 4.

### Recovery and matrix effects

A 50 ng/mL standard (*n* = 3) spiked into BSA (4%) was extracted through a PPT + plate. Recovery of APAP and AT7519 was 53.3 and 56.6% respectively. Matrix effects were calculated as 70.2% for APAP, and 46.5% for AT7519.

### Stability

Standards and QC samples were analysed before and after 48 h at 10 °C in the autosampler and the amount of APAP and AT7519 calculated using the calibration curve from the peak area ratio of the compound/internal standard were compared before and after. Results show that all QC samples met the acceptance criteria for precision and accuracy of 85–115% for %NOM and ± 15% for %CV (Table 5).

**Table 5:** Autosampler stability of APAP and AT7519 in Quality Controls (10 °C, 48 h)

			**APAP**		**AT7519**	
QC level	n	Concentration (ng/mL)	% NOM	% CV	% NOM	% CV
LLOQ QC	4	0.5	97.54	10.00	104.00	1.36
Low QC	4	0.75	105.80	3.14	104.00	1.36
Mid QC	4	5.00	98.03	0.94	97.71	2.10
High QC	4	40.00	95.99	1.28	97.71	1.04

### Mouse Sample Results

The developed method was used to measure levels of AT7519 and APAP in serum from C57Bl6J WT mice that had been treated with either or both drugs. AT7519 was detected in all samples from mice dosed at 10 mg/kg 20 h previously with the drug, both in mice that had received APAP and in mice that had received an APAP vehicle control.

**Fig. 5:**
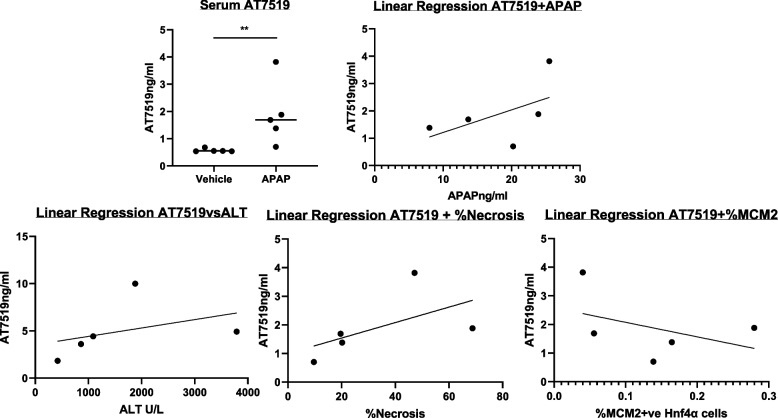
Statistical analysis of AT7519 quantification in mouse serum. **a** Concentrations in mice with and without APAP-ALI. **b** Linear regression analysis of mouse serum AT7519 and APAP (R^2^ = 0.2696), **c** of mouse serum AT7519 and serum ALT (R^2^ = 0.1499), **d** serum AT7519 and % hepatic necrosis (R^2^ = 0.3178), **e** serum AT7519 and hepatic MCM2 expression (R^2^ = 0.1755)

AT7519 levels were significantly higher in mice that had received APAP (4.96, range 1.83–9.99 ng/mL) compared to control (1.50, range 1.41–1.77 ng/mL) (*P* = 0.0079). At this dose of 10 mg/kg there was no correlation between APAP and AT7519 measurement (R^2^ = 0.2696) (Fig. 5). There was no correlation with serum AT7519 concentration and hepatic damage, as assessed by serum ALT and % hepatic necrosis. There was also no correlation of serum AT7519 concentration and hepatocyte proliferation, by quantifying hepatocyte MCM2 expression (Fig. 5).

## Discussion

We have developed and validated a UPLC-MS/MS method to quantify both AT7519 and APAP in mouse serum. The method has improved sensitivity for AT7519 (LLOQ of 0.5 ng/mL) compared to a method used for AT7519 in isolation (LLOQ: 5 ng/mL) [24]. Our method also shows sensitivity improvement on earlier APAP methods applied to mouse samples (LLOQ of 0.5 ng/mL) compared to 0.25 μg/mL in C57Bl6J mouse [28] and 5 ng/mL [29]).

Chromatographic separation and MRM transitions used for detection and quantification of compounds APAP, and AT7519 were optimised carefully for highest ionisation and also chromatographic separation. The mass analyser used in this analysis is a triple quadrupole and for the best sensitivity it is operated in multiple reaction monitoring mode, where an ion is detected in quadrupole 1, fragmented under a defined collision energy and a diagnostic fragment is detected in quadrupole 3. This requires the identification of a parent ion [M + H]^+^, fragmented to give a diagnostic ion – and this is defined as the mass transition. The MRM transitions used for quantitation are chosen by infusing analytical standards and carrying out product ion scans to determine the most appropriate fragment ions. Compound specific voltages for collision energy, declustering potential and collision exit potential and are included in Table 1. For example, for AT7519, which has a molecular weight of 381 Da gave a protonated molecular ion in electrospray ionisation and the (*m/z* 382.1). *m/z* 89.1 was the most dominant transition on the QTrap5500 mass spectrometer, which is consistent with the finding of Dolman et al. For both analytes, APAP and AT7519, isotopically labelled internal standards were used, which enables superlative tracking of analyte retention times on chromatographic separation. This is alongside compensating for various matrix effects that the matrix may introduce to each sample that the internal standard can control for. Inclusion of isotopically labelled internal standards is good practice in targeted LC–MS/MS methods and this has improved upon the first study of isolated AT7519 quantitation, which used the drug Rucaparib as an internal standard and not a labelled version [24].

We assessed our method for dynamic range, determined limits of quantitation, assessed precision and accuracy of quality controls and assessed stability of extracts. Achieving intra and inter-batch QC NOM values within ± 15% of the nominal concentration, and %CV < 15% meets current acceptance criteria for mass spectrometry analysis, along with stability of extracts in typical autosampler conditions For both AT7519 and APAP our method is linear over two orders of magnitude, and has shown to be both precise and accurate, resulting in a reproducible method.

Control mouse serum samples and the surrogate matrix BSA were extracted and analysed using the LC–MS/MS and no peaks with signal to noise ratio of 3 or more were detected at the retention times of APAP or AT7519, demonstrating the specificity of the method. This also shows no evidence that other endogenous substances are eluted at the key chromatographic elution times of either drug. This confirmed that BSA is a suitable surrogate matrix and that both compounds can be measured simultaneously – a novelty of this method.

The recovery and matrix results were considered acceptable, while inclusion of isotopically labelled internal standards for both analytes accounts for any variability of matrix effects that may occur in biological samples. Using BSA as a surrogate matrix offers the opportunity to assess AT7519 and APAP in samples from other species, e.g. rat or human.

The extraction method was developed to use high throughput 96-well automated extraction, which lends itself to high throughput. The run time for this method is 9 min, resulting in an average run time of ~ 15 h per 96-well plate. We assessed autosampler stability over 48 h and found calibration standards and extracts to be exceptionally stable. This reflects real time laboratory conditions, including time for repeat analysis or potential interruption.

The method was sensitive enough to detect AT7519 in only 50 μL mouse serum, 20 h after dosing, which is longer than reported previously. The murine study of Dolman et al. [24], gave i.p. doses of 15 mg/kg AT7519 and found measurable levels of AT7519 in mouse plasma 6 h post dose but undetected in plasma at 24 h. Our study gave a lower dose of 10 mg/kg and was sensitive enough to detect 20 h post dose and in only 50 uL serum. Squires et al. [26] conducted a study which gave 5 mg/kg i.v. injection and detected AT7519 in plasma 6 h post dose, and 16 h post dose in tumour tissue, but longer times were not evaluated.

The lack of correlation between serum APAP and AT7519 quantification after 10 mg/kg dosing, indicates that AT7519 and APAP do not compete metabolically. Bioavailability of AT7519 post i.p. injection has previously been shown to be complete (Squires 2009). The exact mechanism of AT7519 metabolism is unknown, but from these results we can infer that hepatic metabolism is required, as mice with APAP-ALI, which have impaired hepatic function, have higher measurements of the unmetabolized parent compound. This is most likely due to a reduced number of functioning hepatic cells or due to competition with the specific cytochrome p450 enzymes involved in APAP metabolism, e.g. CYP2e1 [30]. However, the lack of correlation between the amount of serum APAP and AT7519 indicates direct competition of CYP2e1 is less likely.

The AT7519 levels in mice with APAP-ALI also indicate that a 10 mg/kg dose of AT7519 does not contribute to hepatic injury or reduce regeneration, by assessing this alongside hepatic damage and proliferation at a repair time point in this model. ALT and necrosis are commonly used markers of hepatic damage both in people and in mouse models of APAP-ALI [15, 31] and they were not correlated with AT7519. MCM2, a key component of cellular pre-replication complex, initiates DNA replication and is used as a marker of cellular proliferation [32–34].

Neutrophils are recruited rapidly to the liver after APAP damage [22, 35]. AT7519 has been shown to resolve inflammation and clear tissue neutrophils in other models [8] and with this additional knowledge: evidence for a lack of AT7519 competition with APAP, and evidence for hepatic metabolism, then additional studies using AT7519 in APAP-ALI models can be completed.

## Conclusion

In this study we report development of a novel UPLC-MS/MS method for the simultaneous quantification of AT7519 and APAP in small volumes of mouse serum (50 μL). The improved sensitivity of this assay for AT7519 compared to previous methods is of benefit for studies using single small doses of AT7519 in mice, which is of particular relevance to inducing granulocyte clearance and resolve inflammation. The additional benefit of being able to assess AT7519 in small volumes of serum alongside APAP ensures optimal utility of available blood and opens the door for adaptation to measure other drug-like compounds simultaneously. At 10 mg/kg AT7519 did not contribute to hepatic injury or reduce regeneration. Our laboratory will now be using this method to assess a higher dose of AT7519 in the context of acute liver injury to assess the response of neutrophil depletion. We will assess if AT7519 modulation of neutrophils can promote the resolution of damaging inflammatory processes.

## Data Availability

The datasets generated and/or analysed during the current study are available in the Edinburgh DataShare repository (10.7488/ds/7453). The datasets are also available from the corresponding author on reasonable request.
