# Correction: Analysis of AT7519 as a pro-resolution compound in an acetaminophen-induced mouse model of acute inflammation by UPLC-MS/MS

**DOI:** 10.1186/s12950-023-00352-z

**Published:** 2023-08-16

**Authors:** Jennifer A. Cartwright, Joanna P. Simpson, Natalie Z. M. Homer, Adriano G. Rossi

**Affiliations:** 1 University of Edinburgh Centre for Inflammation Research, Institute for Regeneration and Repair, 4-5 Little France Drive, Edinburgh BioQuarter, Edinburgh, EH16 4UU Midlothian UK; 2 Centre for Regenerative Medicine, Institute for Regeneration and Repair, University of Edinburgh, 4-5 Little France Drive, Edinburgh BioQuarter, Edinburgh, EH16 4UU Scotland UK; 3 Mass Spectrometry Core, Edinburgh Clinical Research Facility, Centre for Cardiovascular Sciences, Queen’s Medical Research Institute, University of Edinburgh, 47 Little France Crescent, Edinburgh, EH16 4TJ UK

**Journal of Inflammation (2023) 20:20**

10.1186/s12950-023-00345-y

After publication of this article [1], the authors reported that (1) in the ‘Availability of data and materials’-section, the link to the data (MS data (10.7488/ds/7453)) is missing; (2) in Fig. 5, 3rd panel (Linear Regression AT7519 + ALT), the ALT units should be U/L and not µ/l.

The original article [1] has been corrected.
